# Apoptotic resistance of human skin mast cells is mediated by Mcl-1

**DOI:** 10.1038/cddiscovery.2017.48

**Published:** 2017-08-21

**Authors:** Tarek Hazzan, Jürgen Eberle, Margitta Worm, Magda Babina

**Affiliations:** 1 Department of Dermatology and Allergy, Charité Universitätsmedizin Berlin, Charitéplatz 1, Berlin 10117, Germany

## Abstract

Mast cells (MCs) are major effector cells of allergic reactions and contribute to multiple other pathophysiological processes. MCs are long-lived in the tissue microenvironment, in which they matured, but it remains ill-defined how longevity is established by the natural habitat, as research on human MCs chiefly employs cells generated and expanded in culture. In this study, we report that naturally differentiated skin MCs exhibit substantial resilience to cell death with considerable portions surviving up to 3 days in the complete absence of growth factors (GF). This was evidenced by kinetic resolution of membrane alterations (Annexin-V, YoPro), DNA degradation (propidium iodide), mitochondrial membrane disruption (Depsipher), and Caspase-3 activity. Because of the high basal survival, further protection by SCF was modest. Conversely, survival was severely compromised by staurosporine, implying functional caspase machinery. Contrary to the resistance of freshly purified MCs, their culture-expanded counterpart readily underwent cell death upon GF deprivation. Searching for the molecular underpinnings explaining the difference, we identified Mcl-1 as a critical protector. In fact, silencing Mcl-1 by RNAi led to impaired survival in skin MCs *ex vivo*, but not their cultured equivalent. Therefore, MCs matured in the skin have not only higher expression of Mcl-1 than proliferating MCs, but also greater reliance on Mcl-1 for their survival. Collectively, we report that human skin MCs display low susceptibility to cell death through vast expression of Mcl-1, which protects from mortality and may contribute to MC longevity in the tissue.

## Introduction

Mast cells (MCs), strategically located at the interfaces of host and environment, are primary effector cells of IgE-mediated allergic reactions, and thereby contribute to allergic rhinoconjunctivitis, asthma, eczema, urticaria, and in the most severe case, anaphylaxis.^1,2,3^

In the skin, MCs are also associated with itch sensations elicited by immunologic and non-immunologic stimulation.^4,5^

Though of hematopoietic origin, MCs complete their differentiation into mature subsets only after arriving in peripheral organs such as the skin, lung, and gut. MC density is particularly abundant in skin.^6^ The tissue microenvironment shapes the developing MCs, as highlighted by the distinct phenotypes developing in different tissues (broad distinction into MC_T_ and MC_TC_), and it is the tissue-specific niche that also maintains MC survival after completion of their maturation process.^7^

The developmental peculiarity of MCs *vis-à-vis* most other blood-borne cells poses difficulty in obtaining pure, *in situ* differentiated MCs for research purposes. To circumvent the problem, human MC research chiefly employs cells generated in culture from hematopoietic progenitors with no contact to neighboring cells, connective tissue and other elements of their *in vivo* habitat.^8,9,10,11,12^ Our recent research activities within the FANTOM5 consortium (Functional Annotation of the Mammalian Genome), highlighted, however, that tissue-derived MCs progressively lose or modify key lineage attributes upon prolonged culture *vis-à-vis* their counterparts *ex vivo*.^13,14,15^

The regulation of fundamental cell fate decisions, such as death *versus* survival, may likewise experience changes in non-natural surroundings, but such a possibility has to our knowledge not been explored. A difference in survival properties between MC subsets is, however, strengthened by a large body of evidence demonstrating that MCs can differ regarding pro- and anti-apoptotic factors.^16,17,18,19,20,21,22,23,24^

Here, we studied cell death regulation in skin MCs directly *ex vivo*. We report that skin MCs exhibit substantial resistance to cell death even in the complete absence of growth factors (GFs), whereas their cultured counterparts readily undergo cell death. In search of the molecular underpinnings behind the difference, we reveal Mcl-1 as a key factor imparting protection from mortality. Its vast expression within the cutaneous habitat preserves MCs, likely contributing to their longevity.

## Results

### Skin MCs display relative resistance to cell death

Following recommendations from the NCCD (Nomenclature Committee on Cell Death),^25^ there is no single method to unequivocally prove or disprove apoptosis or even general cell death, because each method measures only a certain aspect and can produce false results if viewed in isolation.

To get a first insight into the kinetic order of events after the detachment of MCs from their natural skin habitat, cells were kept in minimal medium in the absence of GF and analyzed for signs of cell death by several methods. The results are specified in the following paragraphs.

#### Cell size

**Figure 1:**
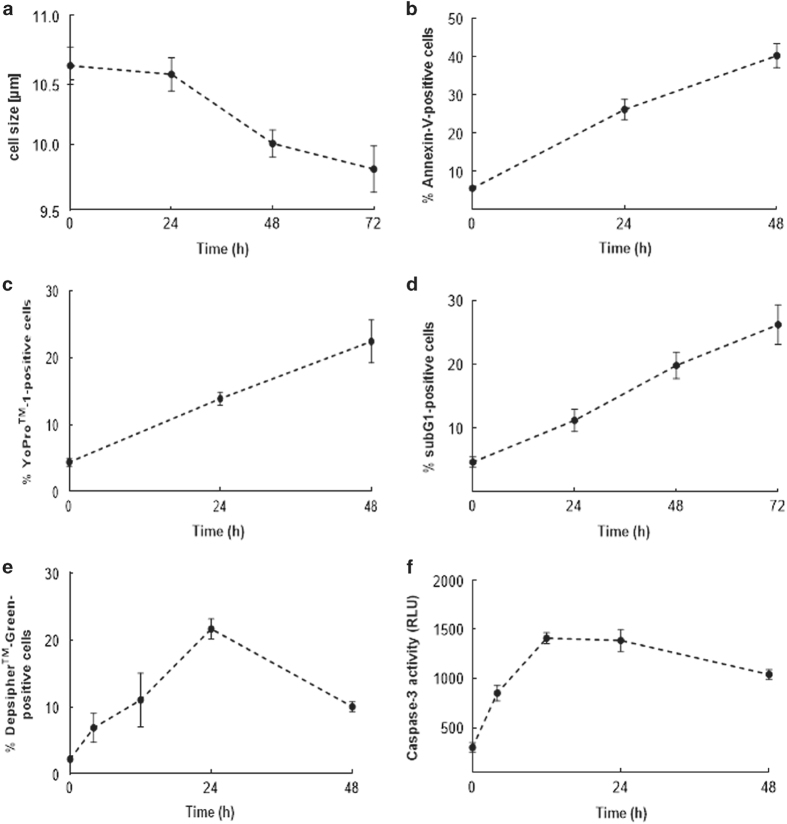
Skin mast cells display relative resistance to cell death. Skin MCs ex vivo were kept in GF/serum-free medium for up to 3 d. (**a**) cell size alteration over time (via automatic cell counter), (**b**–**e**) percent of cells with (**b**) externalized P-Serine (Annexin- V-FITC), (**c**) YoPro positivity, (**d**) fragmented DNA (propidium iodide); (**e**) low mitochondrial membrane potential (Depsipher); (**f**) caspase-3 activity (determined by Caspase-Glo 3/7 assay) RLU=Relative Luminescence Units. Results represent the mean±S.E.M. of at least three independent experiments*.*

Cell shrinkage is more commonly associated with apoptosis, whereas cells undergoing necrotic cell death rather display swelling initially. The mean diameter of a foreskin MC is ≈10.7 *μ*m.^26,27^ We monitored cell size development upon transfer of purified skin MCs to minimal medium. As shown in Figure 1a, MCs slightly, but gradually decreased in size over the 3-day-observation period.

#### Phosphatidylserine (P-Ser) externalization

P-Ser, a phospholipid membrane component, is confined to the inner side of the membrane in healthy cells but can flip to the outer side in apoptotic cells, where it becomes detectable by Annexin-V-FITC binding.^28^ This method has frequently been employed to detect apoptosis in cultured MCs.^10,18,22^

Upon isolation, skin MCs regularly show a high degree of viability (for example, ≈99% trypan blue-negative),^29,30^ as confirmed here by different methods (Supplementary Figure 1).

P-Ser externalization gradually increased over a 48 h observation period, rising to ~25% at 24 h and 40% at 48 h (Figure 1b).

#### Alterations in plasma membrane properties

Apart from P-Ser externalization, apoptotic cells typically undergo further morphologic and biochemical changes in membrane properties.^31^ One method to capture this exploits the fact that the green fluorescent YoPro-1 dye can permeate the slightly porous membrane of apoptotic cells. Thus, the use of YoPro-1/PI enables visualization of P-Ser-independent membrane alterations indicative of apoptosis. Here, the YoPro-1 signal increased time-dependently, and ~20% of the MCs were YoPro-1-positive after 48 h (Figure 1c). Curve progression was comparable to Annexin-V-FITC positivity (Figure 1b *versus* Figure 1c), but the proportion of cells stained by YoPro-1 was typically lower.

#### DNA degradation

We next addressed DNA degradation, a characteristic feature of apoptosis in its later stage,^32^ which can be visualized by propidium iodide (PI) staining of cell nuclei to distinguish between diploid (viable) and sub-diploid (apoptotic) DNA content.

As shown in Figure 1d, nuclei displaying DNA degradation increased in a constant but rather moderate fashion, reaching ~20% after 48 h. Extending the time to 72 h resulted in a further increase to ≈25%.

#### Change in mitochondrial membrane potential

Mitochondria act as central gateway controllers of apoptosis particularly through the release of pro-apoptotic factors into the cytoplasm.^33^ Here, we investigated the electrochemical gradient disruption across the mitochondrial inner membrane, which typically occurs during apoptotic cell death. We used the Depsipher reagent, which aggregates in the mitochondria of healthy cells to form an orange fluorescent compound, whereas it remains in its green form when the MMP (mitochondrial membrane potential) is disrupted.

The ratio of cells displaying MMP disturbance rose progressively for the first 24 h, when it reached a maximum of ≈22%, before declining to ≈10% Depsipher-Green-positive cells after 48 h (Figure 1e).

#### Caspase-3 activity

Kinetic resolution of Caspase-3 activity revealed an increase during the first 12 h, followed by a plateau and eventually a slight decline (Figure 1f).

Collectively, skin MCs *ex vivo* underwent progressive cell death in a time-dependent fashion, and this was proven by different readouts. However, viable MCs were still detectable after 72 h in the complete absence of specific GF and serum, indicating protection of skin MCs from cell death.

### Skin MCs can be induced to undergo rapid apoptosis

The resistance of skin MCs to death, revealed in the previous paragraph, raised the question of whether these cells were susceptible to apoptosis induced by cues like staurosporine, a well-known activator of caspase-mediated cell death.^34,35^

**Figure 2:**
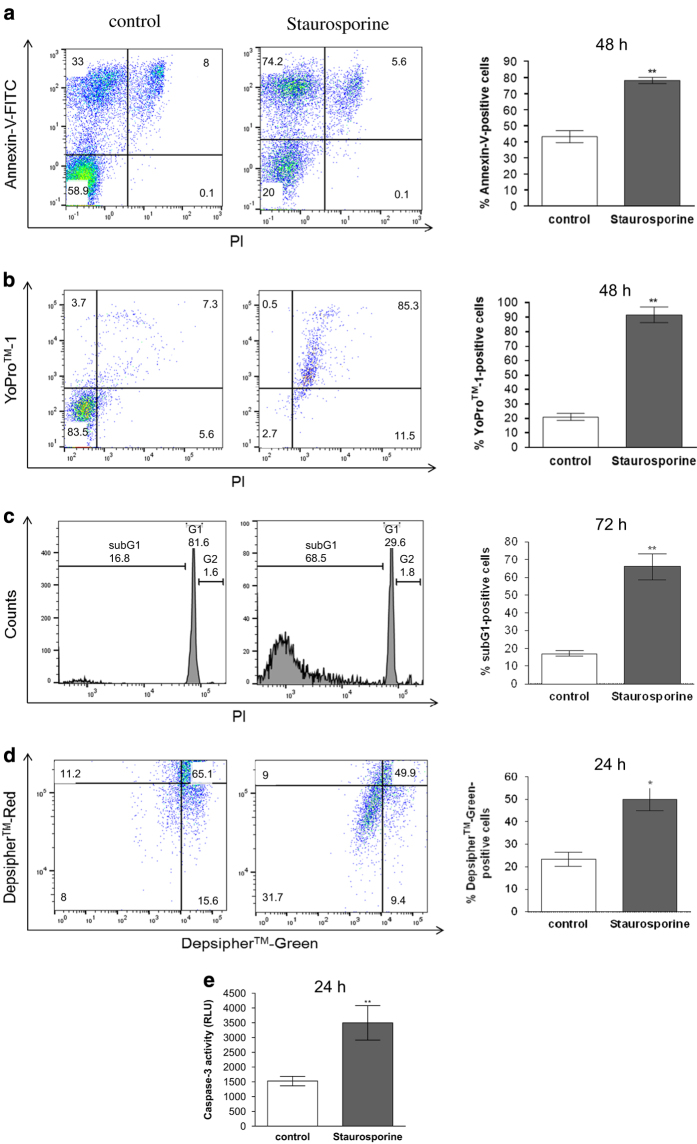
Skin MCs can be induced to undergo rapid apoptosis. Skin MCs ex vivo were kept without or with staurosporine (2 *μ*M) in serum/GF-free medium for the indicated times. (**a**–**d**) percent of cells with (**a**) externalized P-Serine (Annexin-V-FITC), left side: representative flow cytometry dot plots, right side: cumulative data of *n*=5 independent experiments, (**b**) YoPro positivity, left side: representative flow cytometry dot plots, right side: cumulative data of *n*=4 independent experiments, (**c**) fragmented DNA (propidium iodide), left side: representative flow cytometry histograms, right side: cumulative data of *n*=4 independent experiments, (**d**) low mitochondrial membrane potential (Depsipher), left side: representative flow cytometry dot plots; right side: cumulative data of *n*=4 independent experiments; (**e**) caspase-3 activity (determined by Caspase-Glo 3/7 assay), cumulative data of *n*=6 independent experiments, RLU=RLU=Relative Luminescence Units. Results represent the mean±S.E.M. of *n* independent experiments; *P<0.05, **P<0.01.

Indeed, staurosporine led to a huge increment in cell death, and this was largely consistent among detection methods (Figures 2a–e). The proportion of apparently apoptotic MCs not only increased *vis-à-vis* control but also reached remarkable global levels between 50 and 90% by all flow cytometric methods, and there were barely any identifiable MCs left after 72 h. YoPro-1 (Figure 2b) and DNA degradation (Figure 2c) had the highest increments over control, while the effect was less pronounced for Annexin-V-FITC (Figure 2a). Caspase-3, the actual indicator of responsiveness to staurosporine, showed a marked increase in activity (Figure 2e).

Collectively, we found that skin MCs can be forced to undergo cell death, indicating that their apoptotic machinery was intact, but required a potent activator of caspase-mediated death together with GF deprivation to affect a major proportion of the population.

### Stem cell factor (SCF) modestly counters apoptosis of *ex vivo* skin MCs

**Figure 3:**
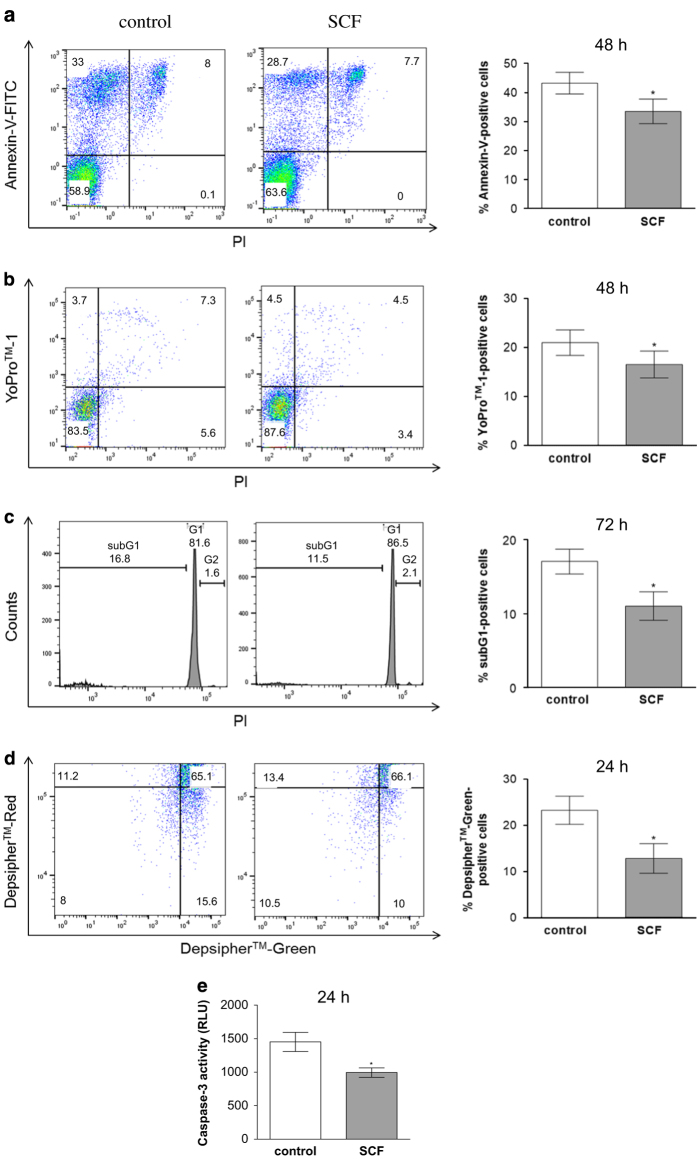
SCF modestly counters apoptosis of ex vivo skin MCs. Skin MCs ex vivo were kept without or with SCF (10 ng/ml) in serum/GF-free medium for the indicated times. (**a**–**d**) percent of cells with (**a**) externalized P-Serine (Annexin-V-FITC), left side: representative flow cytometry dot plots, right side: cumulative data of *n*=4 independent experiments, (**b**) YoPro positivity, left side: representative flow cytometry dot plots, right side: cumulative data of *n*=4 independent experiments, (**c**) fragmented DNA (propidium iodide), left side: representative flow cytometry histograms, right side: cumulative data of *n*=4 independent experiments, (**d**) low mitochondrial membrane potential (Depsipher), left side: representative flow cytometry dot plots, right side: cumulative data of *n*=4 independent experiments; (**e**) caspase-3 activity (determined by Caspase-Glo 3/7 assay), cumulative data of *n*=6 independent experiments, RLU=RLU=Relative Luminescence Units. Results represent the mean±S.E.M. of *n* independent experiments; *P<0.05.

SCF is arguably the most important mediator of MC survival.^36,37^ We tested whether SCF would protect skin MCs from death. As shown in Figure 3, SCF significantly, yet modestly countered MC apoptosis. Again the results were largely consistent among different methods (Figures 3a–e).

SCF was unable to revert the effect of staurosporine when applied before or together with the caspase activator (data not shown), however, suggesting that the effect of staurosporine was dominant.

In summary, our data show that SCF protects skin MCs from cell death without fully maintaining viability.

### Cultured skin-derived MCs are more susceptible to cell death – more pronounced rescue effects from SCF

**Figure 4:**
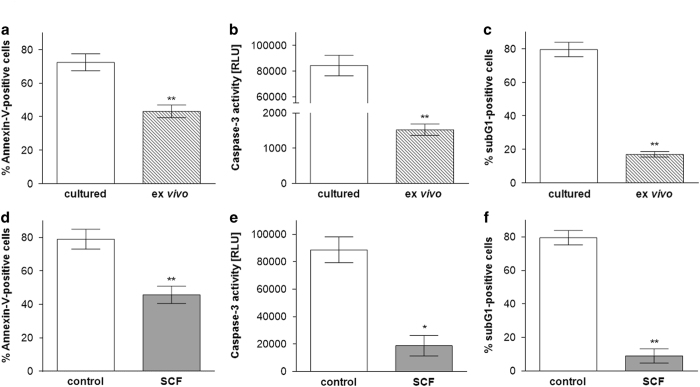
Cultured skin-derived MCs are more susceptible to cell death – more pronounced rescue effects from SCF. (**a**–**c**) Long-term cultured skin-derived MCs were exposed to GF/serum-free medium for a total of 40 h (white columns), *ex vivo* skin MCs, shown for comparison, are as in Figure 2. (**d**–**f**) Long-term cultured skin-derived MCs were pre-cultured in GF/serum-free medium for 16 h and supplied (or not) with SCF (10 ng/ml) for 24 h (SCF-deficient conditions as in **a**–**c**). Percent of cells with (**a**, **d**) externalized P-Serine (Annexin-V-FITC), cumulative data of *n*=4 independent experiments, (**b**, **e**) fragmented DNA (propidium iodide), left side: representative flow cytometry histograms, right side: cumulative data of *n*=4 independent experiments (**c**, **f**) caspase-3 activity (determined by Caspase-Glo 3/7 assay), cumulative data of *n*=4 independent experiments, RLU=Relative Luminescence Units. Results represent the mean±S.E.M. of *n *independent experiments; *P<0.05, **P<0.01.

SCF not only serves as survival factor of human MCs, but it can evoke cell proliferation if used at high concentrations.^26,38^ As expanded skin-derived MCs differ from their *ex vivo* counterparts at multiple levels,^13,14^ we addressed the question of whether *in-vitro* expansion would modify MC susceptibility to cell death, and this was indeed the case. In particular, cultured MCs displayed greater proneness to cell death upon GF deprivation *versus ex vivo* MCs by different methods (Figures 4a–c). Furthermore, the addition of SCF 16 h prior to harvest resulted in a striking decrease in Annexin-V positivity compared with the untreated control (Figure 4d). Accordingly, there was a huge drop in sub-G1-nuclei (Figure 4e) and in caspase-3 activity (Figure 4f) in SCF-treated cells *vis-à-vis* GF-deprived cells. Together, the data suggested greater susceptibility of cultured than fresh skin MCs to apoptosis and a greater rescue effect by SCF in the cultured system.

### Skin MCs *ex vivo* express abundant levels of the anti-apoptotic factor Mcl-1

**Figure 5:**
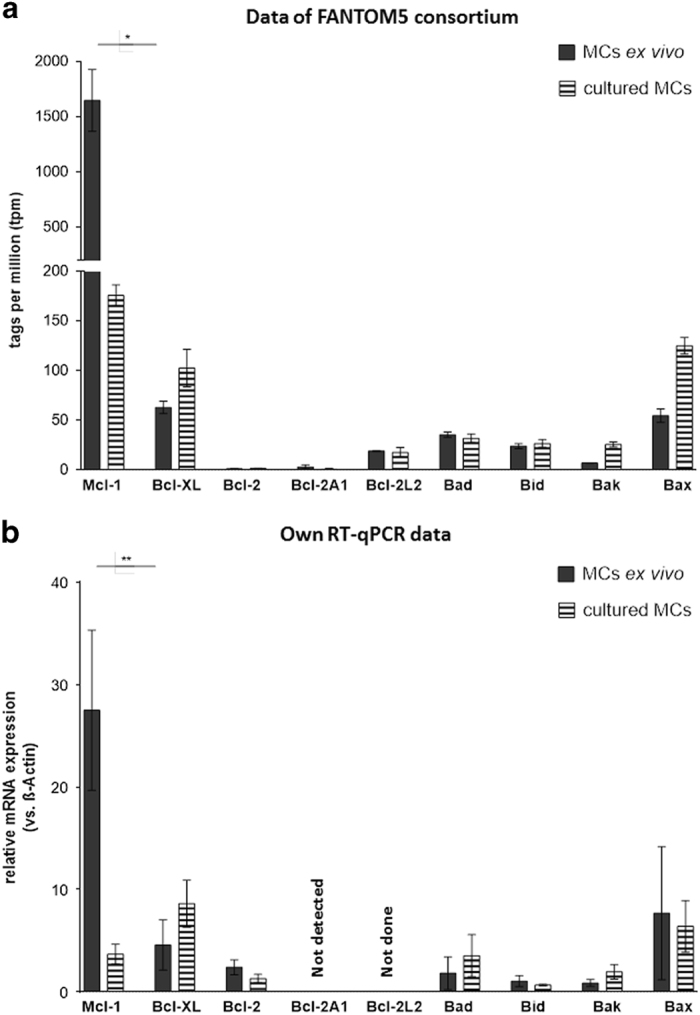
Skin MCs ex vivo express abundant levels of the anti-apoptotic factor Mcl-1. Expression of Bcl-2 family members by human skin-derived MCs ex vivo versus after long-term expansion. (**a**) quantitative deep-CAGE sequencing analysis of mRNA encoding members of the Bcl-2 family, presented in tags per million (from the FANTOM5 expression atlas^12^), (**b**) quantitative RT-qPCR analysis of mRNA encoding members of the Bcl-2 family, normalized to expression of the housekeeping gene *β*-actin, cumulative data of *n*=5 independent experiments. Results represent the mean±S.E.M. of *n* independent experiments; *P<0.05, **P<0.01.

As skin MCs showed less susceptibility to apoptosis than their cultured equivalents (Figures 1b–f *versus* Figures 4a and b), we set out to address the potential protector(s), which impeded or delayed cell death in the natural MC subset. Because Bcl-2 family members are crucially implicated in mitochondria-mediated apoptosis and have been extensively studied in the lineage,^19,21,23,39–41^ we examined their expression in the two MC subsets benefitting from the recently available FANTOM5 expression atlas. The atlas constitutes a comprehensive collection of nearly 1900 promoter-resolved transcriptomes from all sites of the human body, including MCs from our laboratory both *ex vivo* and upon expansion in culture.^13,42^ Of the anti-apoptotic family members Mcl-1, Bcl-x_L_, Bcl-2, Bcl-2a1, and Bcl-2l2, Mcl-1 was most abundant by far, and also most highly differential between *ex vivo* and cultured MCs (9-fold higher in the former), while all other members were comparable between subsets (Figure 5a). The latter was also true for the four pro-apoptotic members of the Bcl-2 family (Bad, Bid, Bak, Bax) tested, although there was a tendency for Bak and Bax to higher expression in cultured MCs (Figure 5a).

Using RT-qPCR, we validated the FANTOM5 deep sequencing data using multiple MC preparations, obtaining a similar pattern (Figure 5b). In particular Mcl-1 was confirmed as the most highly expressed Bcl-2 family member in MCs *ex vivo* in our RT-qPCR panel, and it was likewise the most differential family member among candidates (Figure 5b).

Collectively, the comparative pattern of Bcl-2 family proteins made Mcl-1 the primary candidate to explain the different susceptibility to apoptosis between freshly isolated and long-term cultured skin MCs.

### Mcl-1 imparts resistance of skin MC to cell death

**Figure 6:**
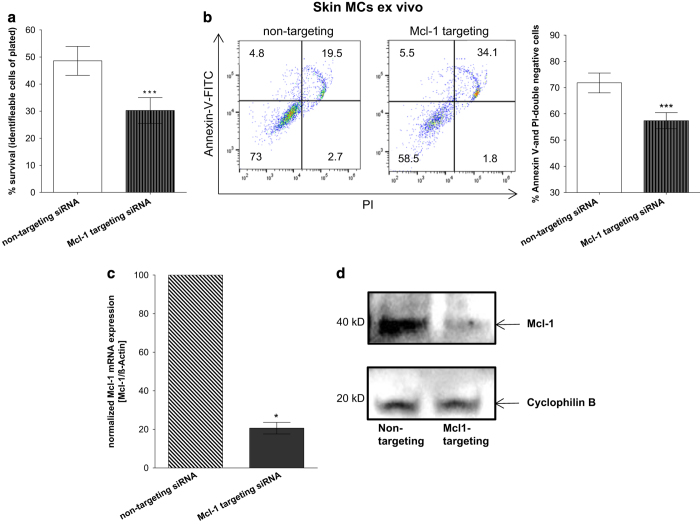
Mcl-1 imparts resistance of skin MC to cell death. Impact of Mcl-1 silencing on MCs after 48 h, evaluated by (**a**) cell number alteration (via automatic cell counter), cumulative data of *n*=6 independent experiments, (**b**) percent of cells with externalized P-Serine (Annexin-V-FITC), left side: representative flow cytometry dot plots, right side: cumulative data of *n*=6 independent experiments, (**c**, **d**) Mcl-1 knockdown confirmation (**c**) on mRNA level by RT-qPCR, cumulative data of *n*=5 independent experiments, (**d**) on protein level by western blot. Results represent the mean±S.E.M. of *n* independent experiments; *P<0.05, ***P<0.005.

Based on the above findings, we directly tested the hypothesis that Mcl-1 was responsible for the resistance of skin MCs to apoptosis. Applying the recently established technique of Accell mediated siRNA self-delivery to skin MCs,^29^ we silenced Mcl-1 and monitored its impact on survival. In fact, RNAi with Mcl-1-targeting siRNA resulted in accelerated cell decline, as evidenced by a drop in MC recovery to only 33% (*versus* 55% upon non-targeting siRNA) (Figure 6a). Accordingly, this was accompanied by a decrease in Annexin-V-FITC/PI-double negative cells upon Mcl-1 knockdown (from 72% in the control to 57% – upon Mcl-1-directed siRNA) (Figure 6b).

Verifying the efficiency of the technique, we found that the Mcl-1-targeting construct indeed resulted in a strong decline in Mcl-1 gene expression compared with non-targeting siRNA (Figure 6c), and this silencing gave rise to robust decline in Mcl-1 protein (Figure 6d).

Collectively, Mcl-1 silencing intensified MC susceptibility to cell death, suggesting a crucial role of Mcl-1 in the protection of skin MCs from mortality.

### Mcl-1 does not significantly contribute to the survival of skin-derived cultured MCs and HMC-1

**Figure 7:**
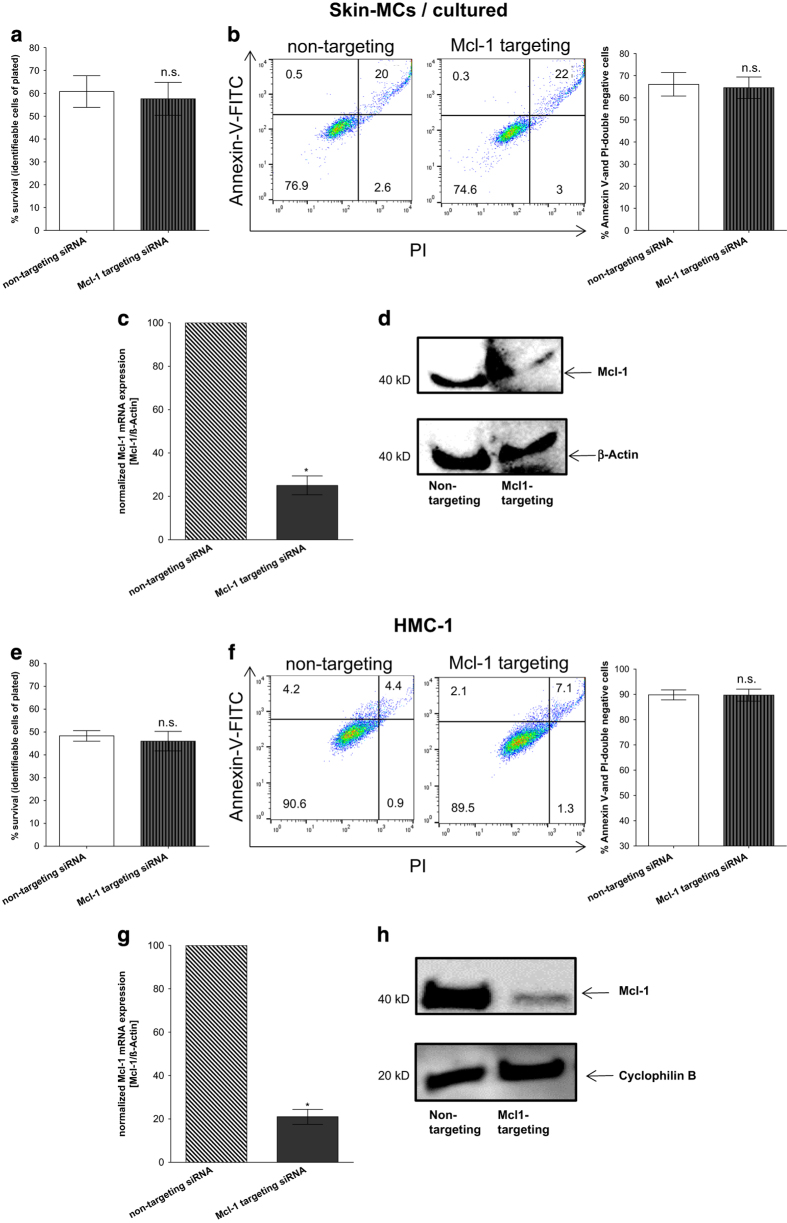
Mcl-1 does not significantly contribute to the survival of skin-derived cultured MCs and HMC-1. Impact of Mcl-1 silencing on (**a**–**d**) cultured skin-derived MCs and (**e**–**h**) HMC-1 after 48 h, evaluated by (**a**,**e**) cell number alteration (via automatic cell counter), cumulative data of *n*=5 independent experiments, (**b**,**f**) percent of cells with externalized P-Serine (Annexin-V-FITC), left side: representative flow cytometry dot plots, right side: cumulative data of *n*=4 independent experiments; Mcl-1 knockdown confirmation (**c**,**g**) on mRNA level by RT-qPCR, cumulative data of *n*=5 independent experiments, (**d**,**h**) on protein level by western blot. Results represent the mean±S.E.M. of *n* independent experiments; n.s.=not significant, *P<0.05.

Since cultured skin-derived MCs displayed lower Mcl-1 expression (see Figure 5) and higher susceptibility to cell death compared with freshly isolated skin MCs (see Figures 4a and b), we hypothesized a minor effect of Mcl-1 in the former subset. Investigating this directly, we found no effect of Mcl-1 siRNA on either cell recovery (Figure 7a) or the proportion of Annexin-V/PI-double negative cells (Figure 7b). The experiments were carried out in the presence of SCF in order to be able to apply the technique (necessity of serum-free conditions).

We further used cells of the Human Mast Cell line-1 (HMC-1) cell line, a malignantly transformed, highly immature MC line^38,43^ as further model of (proliferating) MCs with the result of complete resistance to alterations in Mcl-1 levels (Figures 7e and f).

The lack of effect was not due to lower knockdown efficiency as both cultured skin-derived as well as HMC-1 cells showed the same decrease of Mcl-1 mRNA as skin MCs *ex vivo* (Figures 7c and g *versus* Figure 6c), and this was also true for the respective protein product (Figures 7d and h *versus* Figure 6d).

The combined data indicate that Mcl-1 is not implicated in the maintenance of proliferating MCs, be it skin-derived be it malignantly transformed, in contrast to natural skin MCs.

## Discussion

MCs are believed to be long-lived and can survive even a lifetime, at least in the mouse,^44,45,46^ and there is great interest in the factors establishing this longevity. Therefore, cell death regulation has been studied under a variety of conditions in multiple MC subsets, but no study has to our knowledge addressed the basic fitness of naturally differentiated, tissue-derived human MCs not manipulated by prior culture.

In the present study, we first proved that MCs from human dermis show remarkable survival even in the absence of serum, SCF or any other GF. Protection from cell death was illustrated by combining a variety of techniques, according to guidelines from the NCCD.^47^ The different methods revealed a high degree of congruence, yet with distinct kinetics. The different time-courses were according to expectation because the disruption of the mitochondrial membrane and activation of caspase-3 are viewed as early events, whereas membrane alterations and especially degradation of the DNA occur rather late in the apoptotic cascade.^31,48^ Of the single-cell methods employed, Annexin positivity was most pronounced throughout. This may be explained by the fact that P-Ser externalization is not only a hallmark of apoptosis, but also induced in other scenarios, for example, phagocytosis. Specifically in MCs, P-Ser becomes transiently exposed upon activation.^49^ Therefore, the proportion of Annexin-staining cells may overestimate the real apoptotic proportion. The transient increase in MMP with a rise up to 20% followed by a drop may be explained by the timely elimination of cells displaying disrupted MMP. The same may apply to the plateau observed for caspase-3 activity.

Having uncovered that skin MCs are resistant to cell death even in the absence of GFs, we sought to find the reason behind their remarkable fitness. Making use of staurosporine, which bypasses the early events of apoptosis,^50^ we found that the compound readily induced apoptosis in skin MCs (Figure 2), suggesting that MC health was owed to a blocking event higher in the hierarchy, that is, above caspase activation. This result also clarified that skin MCs are not inherently resistant to apoptosis, for example, by hypoexpression of caspases, but rather protected by (a) factor(s) upstream of caspases.

SCF is arguably the most important GF of the lineage, influencing nearly all aspects of MC biology, including chemotaxis, adhesion, phenotype, mediator production, and stimulability by Fc*ε*RI aggregation, as it also potently counters MC death.^36,51^ When investigating whether SCF prolonged skin MC survival, we indeed found a protective yet rather modest effect (Figure 3), probably because baseline survival was already pronounced.

SCF is indispensable for culture of non-transformed human MCs, and can even trigger proliferation at supra-physiological concentrations, leading to their expansion.^13,14,26,52^ We tested whether such skin-derived, but cultured MCs would react more vigorously to SCF (after GF deprivation), and this was clearly the case (Figure 4). SCF’s potent anti-apoptotic effect detected for cultured MCs is in accordance with multiple earlier studies with different types of cultured MCs, where absence of survival factors provided the impetus for suicide, while swift provision of SCF rescued MCs from mortality.^18,20,37^

Strikingly, however, cultured MCs did not only show greater dependence on SCF for survival, they also displayed a heightened proneness to cell death altogether (Figure 4). This difference could be excellently exploited to pinpoint the factor(s) maintaining survival of *ex vivo* MC. From the impact of staurosporine it was clear that MCs are able to undergo apoptosis if caspases are activated, so that the block was expected to lie higher up in the hierarchy. Bcl-2 family members are the key players regulating the mitochondrial pathway of apoptosis.^19,21,23^ We screened the FANTOM5 expression atlas for differences between skin MCs *ex vivo* and after culture.^13^

The majority of family members were either not differential or showed minor expression only, for example, Bcl-2 itself. Conversely, Mcl-1 was revealed as the primary candidate to explain the difference. It was abundantly expressed in MCs *ex vivo*, but much less so in cultured MCs. It was also the most highly expressed family member whatsoever. Validation of the deep sequencing data by RT-qPCR rendered comparable results, where Mcl-1 was also the only gene to significantly differ between the subsets. Therefore, Mcl-1 seemed an ideal candidate to explain the robustness of freshly isolated MCs.

To evidence a role for Mcl-1, we employed the newly established technique for gene knockdown in skin MCs,^29^ a strategy that proved that Mcl-1 was in fact imparting protection to skin MCs, thus aiding in their maintenance. In contrast, it was of substantially less significance to both cultured MC subsets employed. We conclude that *in vivo* matured, that is, natural skin MCs have both higher expression of Mcl-1 than their cultured counterparts and greater reliance on Mcl-1 for their survival.

Our current data fit the concept that death/survival decisions are mediated by different sets of Bcl-2 members in each MC subset. For example, cultured human MCs (generated from precursors or skin-derived) seem to require Bcl-x_L_ for survival,^24^ whereas necessities likewise differ between murine MMC- and CTMC-like cells.^53^ Because essential roles of Bcl-2 and Bcl-x_L_ were found in several studies for cultured, proliferating MCs,^19,41^ we may speculate that Bcl-2 and Bcl-x_L_ have essential roles in the process of MC formation,^54^ as well as in proliferative MCs,^19,40^ whereas their roles vanish once MCs have completed differentiation and become quiescent in terms of cell cycle progression.

Support for this theory comes from evidence in the literature showing dependence of other non-cycling immune cells on Mcl-1. An important example is long-lived plasma cells, for which Mcl-1 was uncovered as the dominant survival factor, while plasma cell precursors more strongly depended on Bcl-2 and Bcl-x_L_ for survival.^55^

In FANTOM5 highest expression of Mcl-1, in addition to (*ex vivo*) skin MCs, was detected in granulocytes (neutrophils, eosinophils, basophils), which are end-stage differentiation cells, and do not proliferate. In fact, an important role of Mcl-1 in the prolongation of neutrophil survival has been extensively documented and reviewed,^56^ further supporting the notion that Mcl-1 is a major contributor to survival of hematopoietic cells after their exit from the cell cycle. In analogy to these other leukocytes, we surmise that the completion of MC differentiation in skin depends on the accumulation of Mcl-1, whereas MCs at proliferative stages depend less strongly on this factor. Mcl-1 function indeed depends on cellular context and its knockdown can even increase sensitivity to apoptosis triggered by the extrinsic route in tumor cells.^3^

Interestingly, under circumstances of lysosomal destabilization, MCs seem to be prone to apoptotic death.^57,58^ The pathway may even preferentially occur in the lineage owing to its high content of serglycin proteoglycans, yet it requires secretory granule permeabilization and protease leakage into the cytoplasm as a trigger. It does not seem to be spontaneously activated under homeostatic conditions, because MCs are long-lived in their natural environment. In fact, elegant early studies revealed that MC survival in skin was substantially prolonged *vis-à-vis* other leukocytes ^45,46^ even though it remained unknown, how longevity is established. Our present data imply that in the absence of lysosomal disruption,^57,58^ it is the accumulation of Mcl-1 that, at least in part, bestows this quality.

In summary, we document that human skin MCs are equipped with baseline resistance to cell death, surviving even harsh environmental conditions. Therefore, the skin provides a milieu that supports the persistence of MCs, whereas liquid culture, despite supra-physiological concentrations of SCF, does not fully mimic this micromilieu and gives rise to MCs with altered properties.^13,14^ Our results identify a critical role for Mcl-1 in the maintenance of skin MCs. Forcing MCs into the cell cycle by saturating amounts of SCF increases proneness to cell death, and concurrent rescue by SCF as it likewise diminishes Mcl-1 expression and eliminates their dependence on this anti-apoptotic Bcl-2 member. We propose that skin MC longevity, established by the MC-supportive niche of the dermis surpasses SCF, and requires robust up-regulation of Mcl-1, which actively suppresses MC demise.

## Materials and methods

### Purification of human skin MCs

MCs were isolated from human foreskin (circumcision), where typically material from several donors was combined for one experiment. MCs from individual donors were also used for confirmatory purposes for the majority of methods. The purification was performed using an optimized and frequently employed protocol.^13,27,30,38^^,38^

In brief, human skin was cut into strips and treated with dispase (BD Biosciences, Heidelberg, Germany) at 3.5 U/ml and 4 °C overnight. After removal of the epidermis, the dermis was chopped into small pieces and digested with collagenase type 1 at 10 mg/ml (Worthington, Lakewood, NJ, USA) for 1 h at 37 ° C. MC purification was achieved by positive selection with mouse anti-human c-Kit-coated microbeads and an Auto-MACS separation device (both from Miltenyi Biotec, Bergisch Gladbach, Germany). MC purity consistently exceeded 98%, as assessed by acidic toluidine-blue staining (0.1% in 0.5 N HCl).^29,30^ Viability by trypan blue exclusion was >99% and by flow cytometric methods between 93 and 95% (Supplementary Figures A–C).

### Determination of cell number and size

Cells were diluted 1:200 with Casy buffer and cell counting was accomplished by the means of an automatic cell counter and analyzer (Casy Model TTC, Roche/Innovatis, Mannheim, Germany). Mean cell diameter was quantified with the same device.

Acquisition of particle diameter allowed distinguishing between cells with damaged and intact membrane. The latter were considered as viable cells, and the cell number of this fraction served to determine MC survival in % according to the following formula (final cell count/plated cell count)×100.

### Cell treatment

Skin MCs (~10^6^ cells/well) were kept in minimal medium consisting of Basal Iscove medium (with stable glutamine; Biochrom, Berlin, Germany), supplemented with 0.5% BSA (Serva, Heidelberg, Germany). MCs were left untreated or stimulated with SCF (Peprotech, Rocky Hill, CT, USA) (at 10 nM) or treated with staurosporine (Enzo Life Sciences, Lörrach, Germany) (at 2 *μ*M) for the indicated times. After incubation, cells were washed with 1×Dulbecco's phosphate-buffered saline (DPBS) (Thermo Fisher Science, Berlin, Germany) and processed for downstream applications (as described below).

### Long-term MC culture

For generating culture-expanded skin-derived MCs, freshly isolated skin MCs (~5×10^5^ cells/ml) were incubated in Basal Iscove medium, supplemented with 10% FCS (Biochrom) and SCF (Peprotech) (at 100 ng/ml) to evoke proliferation. Cell proliferation typically started after 6–8 weeks of culture.^26,38,52^

The HMC-1 (kindly provided by Dr JH Butterfield) was grown in Basal Iscove medium, supplemented with 10% FCS.

### Flow cytometric analysis of survival

Survival of human skin MCs were examined at the indicated times using several flow cytometry based methods (described below). The stained cells were measured on a MACS Quant FACS (Miltenyi Biotec) and analyzed using the FlowJo software (FlowJo LLC, Ashland, OR, USA).

#### 2.3.1. Annexin-V-FITC

Phosphadidylserine externalization was assessed using an Annexin-V-FITC Apoptosis detection kit (eBioscience, San Diego, CA, USA) according to the manufacturer’s instructions.

By double-staining with Annexin-V-FITC and PI, subsets of cells that were Annexin-V-positive and PI-negative (indicative of apoptosis) or Annexin-V-positive and PI-positive (suggesting necrotic and/or cells in advanced apoptosis) were determined. Annexin-V-FITC and PI-double negative cells were regarded as living.^33^

#### YoPro-1

Membrane porosity was examined using the Membrane Permeability/Dead Cell Apoptosis Kit with *YoPro-1* and PI (Invitrogen, Paisley, UK) in accordance with the manufacturer’s instructions. Double-positive cells were considered necrotic and/or cells in advanced apoptosis, whereas *YoPro-1*-positive and PI-negative cells were regarded as apoptotic cells.

#### Propidium iodide

The percent of hypodiploid nuclei corresponding to cells with fragmented DNA were determined by flow cytometric analysis after PI DNA staining. In brief, cells were stained in 40 *μ*g/ml PI (SigmaAldrich, Taufkirchen, Germany), 0.1% sodium citrate and 0.1% triton X-100 for 1 h at 4 °C and analyzed by flow cytometry. Sub-G1 cell fractions correspond to cells with fragmented DNA (indicating apoptosis).^59,60^

#### Depsipher MMP

The mitochondrial membrane potential was evaluated using Depsipher fluorescent staining (R&D systems, Wiesbaden, Germany) according to the manufacturer’s instructions, where slightly Depsipher-Green-positive and highly Depsipher^-^Green-positive cells are considered apoptotic, whereas Depsipher-Red-positive and Depsipher-Red/Green-double-positive cells are considered viable.

### Caspase-3 activity

Caspase-3 activity of MCs was detected, using a luminometric assay kit (Caspase-Glo 3/7; Promega, Mannheim, Germany) according to the manufacturer’s instructions. The assay provides a proluminescent caspase-3/7 substrate, which contains the sequence DEVD that is cleaved to release luminescence. The light detection was performed by means of a microplate reader (Perkin Elmer, Berlin, Germany).

### RT-quantitative PCR

RT-qPCR was performed as described.^38^ In brief, total RNA was isolated using the Nucleo spin RNA Kit (Macherey-Nagel, Düren, Germany), and RT-qPCR was carried out with the LC Fast Start DNA Master SYBR Green kit (Roche Applied-Science, Basel, Switzerland). The oligonucleotide primers (TIB Molbiol, Berlin, Germany) were as follows:
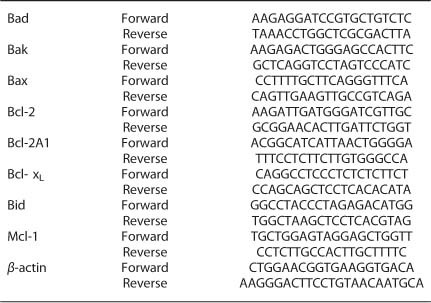


The expression levels of the target gene were quantified relative to the expression of the reference gene *β*-actin using the 2-ΔΔCT method.

### siRNA transfection

RNA interference in MCs was performed according to a recently established protocol^29^ using the Accell siRNA Transfection Kit (Dharmacon Accell siRNA, GE Healthcare Dharmacon, Lafayette, CO, USA), where MCs were transfected by gene-targeting siRNA or non-targeting siRNA. In brief, the cells (~10^6^ cells/well) were washed with 1×Accell siRNA Buffer and then resuspended in Accell siRNA Delivery Medium and added to Accell siRNA Delivery Medium containing 2 *μ*M siRNA (Mcl-1-targeting or non-targeting) for a final concentration of 1 *μ*M and then incubated for 48 h. In case of culture-expanded MCs, the transfection was carried out in the presence of SCF (10 ng/ml). After incubation, cells were washed with 1× DPBS (Thermo Fisher Science) and processed for downstream applications (see RT-qPCR, immunoblotting, cell counting and Annexin-V-FITC staining).

### Immunoblotting

MCs were lysed and separated through 12% SDS-PAGE.^29,38^ After electrophoresis, the proteins were transferred to nitrocellulose membranes. The membranes were blocked with 1× Casein Blocking Buffer (Sigma Aldrich, St Louis, MO, USA) and incubated with primary (anti-Mcl-1 and anti-*β*-actin, each diluted 1 : 1000) antibodies (all from Cell Signaling Technologies, Danvers, MA, USA) overnight and subsequently with (1 : 20 000 diluted) HRP (horseradish peroxidase)-conjugated secondary antibodies (Merck Millipore, Darmstadt, Germany) for 1.5 h. Finally, blots were visualized by a chemiluminesence assay (Weststar Ultra 2.0, Cyanagen, Bologna, Italy) according to the manufacturer’s instructions, and the bands were recorded using a detector for chemiluminesence (Fusion FX7 Spectra, Vilber Lourmat, Eberhardzell, Germany).

### Statistical analysis

Results are reported as mean±standard error of the mean (S.E.M.). Data were statistically analyzed by the paired *t*-test (Figures 2, 3, 4a, 5b and 6c, d). The comparison of Annexin-V positivity and caspase-3 activity in *ex vivo* MCs *versus* cultured MCs was analyzed by the unpaired *t*-test (Figures 4a and b). For the comparison of Mcl-1 mRNA expression in *ex vivo* MCs *versus* cultured MCs the unpaired *t*-test was applied (Figure 5a). For normalized data the Wilcoxon matched-pairs signed rank test was used (Figures 6a, 7a and e). *P*-values<0.05 were considered statistically significant. Data were analyzed with GraphPad Prism Version 6.01 Software (San Diego, CA, USA).

## Publisher’s note

Springer Nature remains neutral with regard to jurisdictional claims in published maps and institutional affiliations.
