# Supplementary material for: Apoptotic resistance of human skin mast cells is mediated by Mcl-1
Source: Cell Death Discov. 2017 Aug 21;3:17048–. doi: 10.1038/cddiscovery.2017.48 (PMC5563844; doi:10.1038/cddiscovery.2017.48)
Supplement: Supplementary Figure 1 Legend [file cddiscovery201748-s1.docx]

*Suppl. Figure 1: Flow cytometric assessment of mast cell viability immediately after isolation from human skin, as detected by* ***A)*** *Annexin-V-FITC / PI staining (double-negative cells indicate vital cells; representative flow cytometry dot plot),* ***B)*** *YoPro^TM^-1 / PI staining (double-negative cells indicate vital cells, a representative flow cytometry dot plot is shown) and* ***C)*** *Propidium iodide staining, the G1/(S)/G2 cell fractions indicate vital cells, while sub-G1 cells have fragmented DNA indicative of apoptosis; a representative flow cytometry histogram is shown.*
