# Supplementary figures and images for: Apoptotic resistance of human skin mast cells is mediated by Mcl-1
Source: Cell Death Discov. 2017 Aug 21;3:17048–. doi: 10.1038/cddiscovery.2017.48 (PMC5563844; doi:10.1038/cddiscovery.2017.48)

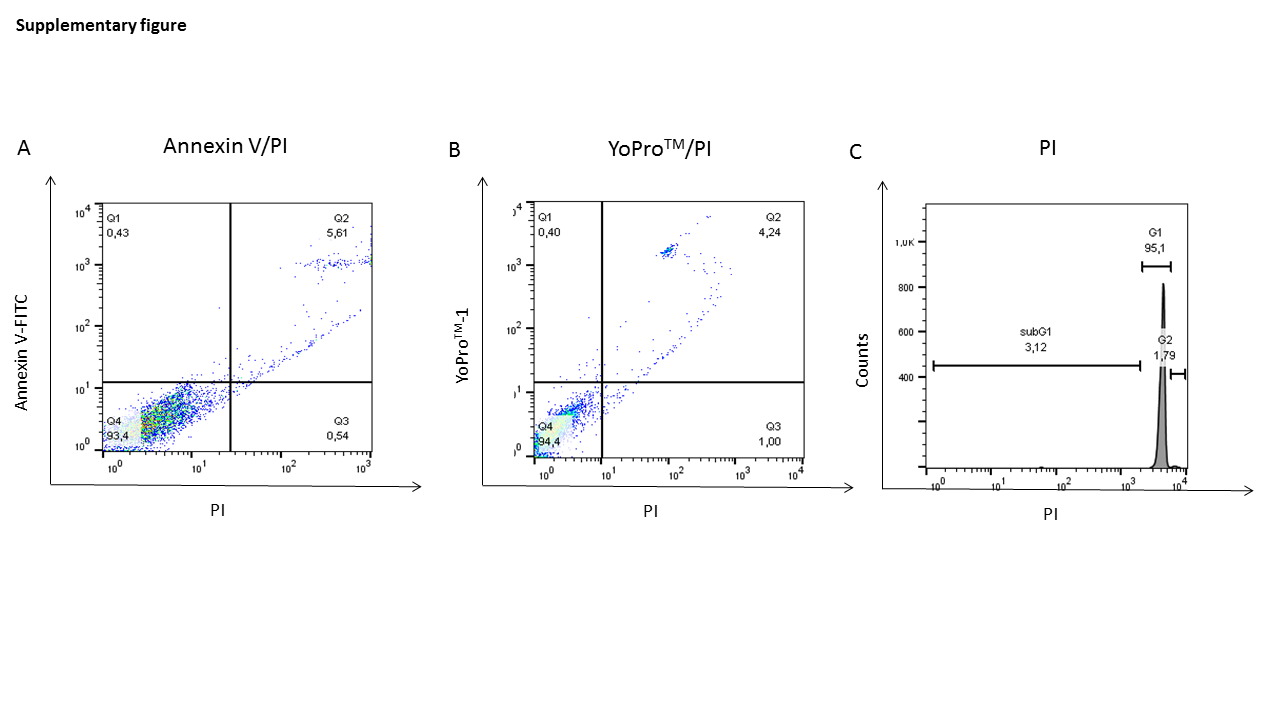

Supplement: Supplementary Figure [file cddiscovery201748-s2.tiff]
